# Breeding and Economic Aspects of Cytogenetic Screening Studies of Pigs Qualified for Reproduction

**DOI:** 10.3390/ani10071200

**Published:** 2020-07-15

**Authors:** Barbara Danielak-Czech, Anna Kozubska-Sobocińska, Grzegorz Smołucha, Marek Babicz

**Affiliations:** 1 Department of Animal Molecular Biology, National Research Institute of Animal Production, Krakowska 1, 32-083 Balice n. Kraków, Poland; barbara.czech@izoo.krakow.pl (B.D.-C.); anna.sobocinska@izoo.krakow.pl (A.K.-S.); 2 Institute of Animal Breeding and Biodiversity Conservation, Faculty of Biology, Animal Sciences and Bioeconomy, University of Life Sciences in Lublin, Akademicka 13, 20-950 Lublin, Poland; marek.babicz@up.lublin.pl

**Keywords:** pigs, fertility, karyotype abnormalities, cytogenetic screening studies

## Abstract

**Simple Summary:**

The cytogenetic screening of pigs, carried out using continually refined cytomolecular techniques, enables a precise diagnosis of chromosomal abnormalities that cause developmental anomalies and considerably reduce the fertility (by several dozen to 100%) and performance parameters of breeding herds, resulting in substantial financial losses. Due to the potential spontaneous occurrence of chromosomal aberrations and the rapid spread of these genetic defects in the population, especially under artificial insemination conditions, it is necessary to perform cytogenetic monitoring of animals qualified for reproduction, which is an important criterion when formulating specific selection guidelines.

**Abstract:**

Cytogenetic monitoring allows the identification and early removal of pigs affected by inherited karyotype defects from breeding herds. These abnormalities cause developmental anomalies, considerably reducing the fertility (by several dozen to 100%) and performance parameters of breeding herds, resulting in substantial financial losses. This mainly concerns reciprocal translocations, typical of pigs, which are highly prevalent (about 0.46%), generally occur de novo, and normally result in low breeding soundness of the carriers. Due to the potential spontaneous occurrence of chromosomal aberrations and the rapid spread of these genetic defects in the population, especially under artificial insemination conditions, it is necessary to perform routine karyotype screening of animals qualified for reproduction. The cytogenetic screening program for young boars, carried out using continually refined diagnostic techniques, permits a precise and reliable karyotype assessment, identification of chromosomal abnormalities, and formulation of specific selection guidelines.

## 1. Introduction

One of the major issues facing pig breeders is karyotype defects, which considerably reduce fertility parameters (by several dozen to 100%) and thus the productivity of breeding herds, resulting in substantial financial losses. These anomalies are generally heritable, occur spontaneously in animals with normal conformation (and semen parameters), and their hidden nature allows them to spread rapidly in populations, especially through artificial insemination [1,2,3]. These factors justify the necessity of routine karyotype screening of pigs qualified for reproduction [4].

Reliable assessment of the porcine chromosome set and detailed identification of abnormalities are based on more and more precise research techniques. This allows the early elimination of animals carrying aberrations from breeding herds. These aberrations are most often reciprocal translocations, typical of pigs, which generally occur de novo, are highly frequent (about 0.46% in populations of cytogenetically monitored boars qualified for reproduction), and normally lead to low breeding soundness of the carriers [1,5,6,7,8,9,10,11]. It should be noted that in practice, cytogenetic analysis showing a normal karyotype provides breeders with an additional criterion to qualify sows and boars for reproduction [2,4,12,13].

The application of this indicator in selection is favored by the fact that the global costs of cytogenetic monitoring of breeding stock are distinctly lower than the financial losses connected with the use of sires with chromosomal defects for breeding. As no comparative analyses in this regard have been performed in recent years, these conclusions are formulated based on French estimates, which account for the costs of karyotype screening and the economic consequences of using boars carrying reciprocal translocations in AI (artificial insemination) stations [4]. Considering the incidence of reciprocal translocations, which was determined for the group of sires qualified for reproduction as 1/200, and taking into account that one cytogenetic analysis costs about 60 euros, the cost of identifying a karyotype defect was calculated to be about 12,000 euros (200 × 60, where 60 euros is the cost of a single karyotype analysis) [4]. In contrast, the global cost of using a translocation-carrying boar in an AI station is about 20,000 euros (calculated using the actual reproductive period of the translocation-carrying boar until it is diagnosed with reduced fertility, which is determined from its mating results; not earlier than after 4 months). During this period, such an animal will produce about 160 litters (40 litters per month), which means that the number of piglets not obtained over the 4-month reproductive period will total 640 (160 × 4, where 4 is the average reduction in the number of piglets per litter as a result of carrying the translocation) [4]. This causes breeders a loss of 19,200 euros (640 × 30, where 30 euros is the price of one piglet). Financial losses are much higher when the reciprocal translocation is spread by a purebred boar from the selection or multiplication levels of the production pyramid because 50% of his offspring will, again, carry this heritable chromosomal mutation [4].

The economic calculation presented above is a concrete argument for pig breeding organizations and breeders associations to systematically eliminate carriers of chromosomal anomalies from the population. This calculation also confirms the need for routine cytogenetic screening of breeding stock as part of the genetic improvement programs of breeds and lines to improve their fertility, which largely determines the economic efficiency of breeding herds. The highest effectiveness of these activities will be ensured by the early prevention of cytogenetic defects based on the general principle of qualifying those young animals for breeding herds, which are screened for karyotype normality before their reproductive use [4,12]. In some countries, identification of chromosomal aberration carriers and their elimination from breeding is an established standard or a legal obligation arising from the implementation of tasks related to the organization of farm animal breeding and reproduction and from sire evaluation and selection programs to ensure planned breeding progress [13,14,15,16].

## 2. Pig Karyotype Abnormalities and Their Effect on Carrier Fertility

The domestic pig karyotype is characterized by a tendency for an increased incidence of heritable, balanced structural chromosomal rearrangements such as reciprocal, Robertsonian, and tandem translocations, as well as paracentric and pericentric inversions. They are caused by spontaneous breakages of unstable chromosomal regions induced by adverse environmental factors [17,18]. These aberrations alter chromosome morphology (generally without loss of genetic material and without phenotypic changes), and their carriers show poorer reproductive performance compared to the herd average [1,19]. This results from errors in the meiotic division during gametogenesis, in which, apart from bivalents, atypical conjugation structures with chromosomes involved in the aberration (tetravalent, trivalent, inversion loop) are formed. This determines a mutation-specific model for the segregation of genetic material into nascent oocytes or spermatozoa. As a result, apart from normal and genetically balanced germ cells, various proportions of gametes with unbalanced genetic (aneuploid gametes) are formed. After their fertilization, they form, respectively, embryos with a normal or balanced karyotype (which means that the defect is inherited by 50% of the offspring) and embryos with an unbalanced chromosome set, which die early in development (as reflected in the lower number of piglets per litter) [2,20,21,22,23,24].

### 2.1. Reciprocal Translocations

The most prevalent type of structural rearrangements in pigs are reciprocal translocations, which are caused by the exchange of chromatid parts between two or more nonhomologous chromosomes. These heritable chromosomal mutations affect all autosomal chromosomes and sex chromosomes, and among almost 200 translocations that have been identified to date, no two identical cases have been found in unrelated animals [1,2,4,12,13,15,16,17,18,19,25,26,27,28,29,30,31,32,33,34,35,36]. The individual consequences of each translocation depend on the morphology of the chromosomes involved, size of the rearranged fragments, and location of centromere breakpoints and positions as factors determining the gametogenesis process (meiotic conjugation and segregation) and the proportion of aneuploid gametes produced (about 40% on average). Ultimately, this determines the real extent of the adverse consequences of carrying these defects, which is assessed based on a reduction in the mean number of piglets per litter (from 5% to 100%). Given that reciprocal translocations are an important reason for the reduced fertility of sires and herds, they should be regarded as a major breeding problem [1,19,22].

### 2.2. Robertsonian Translocations and Tandem Fusions

Another category of karyotype defects is Robertsonian translocations and tandem fusions, which are rare or sporadic in pigs (just a few cases of translocations involving acrocentric chromosome pairs 13, 14, 15, 17, and 18, and one case of tandem-fusion translocation involving chromosome pairs 14 and 17) [1,2,4,12,13,16,19,35,37,38,39,40,41,42,43,44,45,46,47]. Robertsonian translocations always result from the fusion of acrocentric chromosomes in the pericentromeric region, whereas tandem fusions are initiated by fusions in the peritelomeric and pericentromeric regions. A direct outcome of such karyotype rearrangements is the loss of centrometic or telomeric regions, the formation of a large biarmed chromosome, and a reduction in the number of chromosomes. The gametogenesis process, due to asymmetric segregation of the trivalent, results in the production of aneuploid gametes (10% on average), which, after fertilization, give rise to embryos that die early in development, resulting in a small (about 5–22%) increase in infertility. The other 90% genetically balanced or normal gametes give rise to embryos capable of further development, including translocation carriers that transmit this defect to subsequent generations [1,19,22].

### 2.3. Peri- and Paracentric Inversions

Another type of structural karyotype rearrangements is peri- and paracentric inversions, which are relatively infrequent (0.06%) in pigs (more than 20 cases, including a recurrent inversion of chromosome 4) [1,2,4,12,13,16,19,21,35,38,41,48,49]. In these inversions, a chromosome segment derived from two chromatid breaks is turned 180°, leading to asymmetric segregation and unbalanced genetic material in a small portion of the produced gametes, embryos, and fetuses (about 4%), which in the postnatal period may result in a slight (several percent) reduction in fertility. In general, however, inversions cause no gametogenesis or embryogenesis disturbances, and the resulting individuals have a normal or balanced karyotype (at 50% ratio), with the latter spreading these mutations in large populations [1,19,22].

### 2.4. Sex Chromosome Aneuploidies and Leukocytic Chimerism

Apart from balanced chromosome mutations, pigs are sporadically diagnosed with sex chromosome aneuploidies (mainly in a mosaic form) [13,50,51,52,53], and phenotypically normal boars or individuals with hermaphroditic characteristics are increasingly diagnosed with leukocytic chimerism (XX/XY) [1,3,13,19,22,54,55,56,57,58].

## 3. Cytomolecular Diagnostics

**Figure 1:**
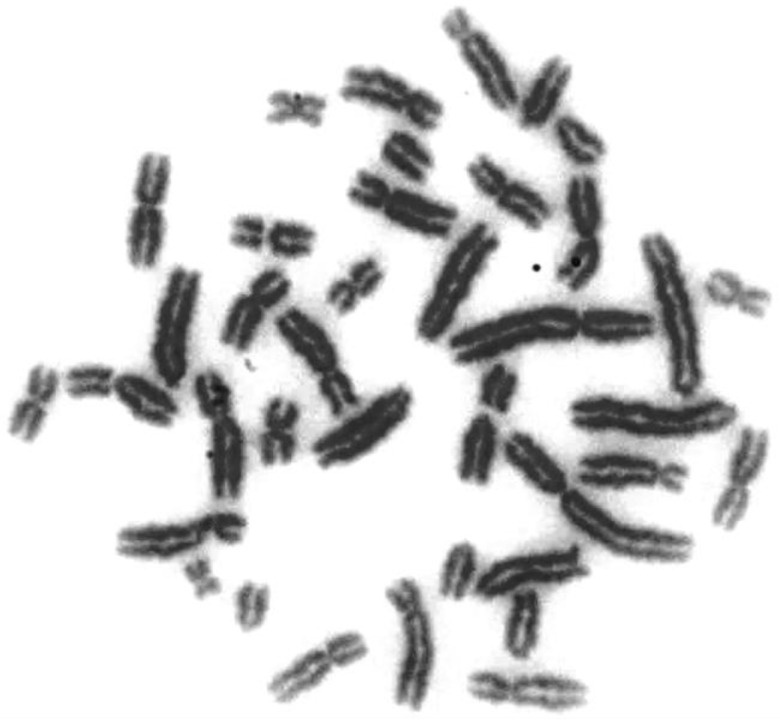
Metaphase chromosomes of boar with 38 XY karyotype-conventional Giemsa staining.

Conventional cytogenetic diagnostics are based on the microscopic analysis of metaphase chromosomes obtained from in vitro lymphocyte culture that is nondifferentially stained with Giemsa, which allows the determination of the diploid number (2*n* = 38) and morphology of chromosomes (submetacentric, metacentric, subtelocentric, and telocentric) (Figure 1).

**Figure 2:**
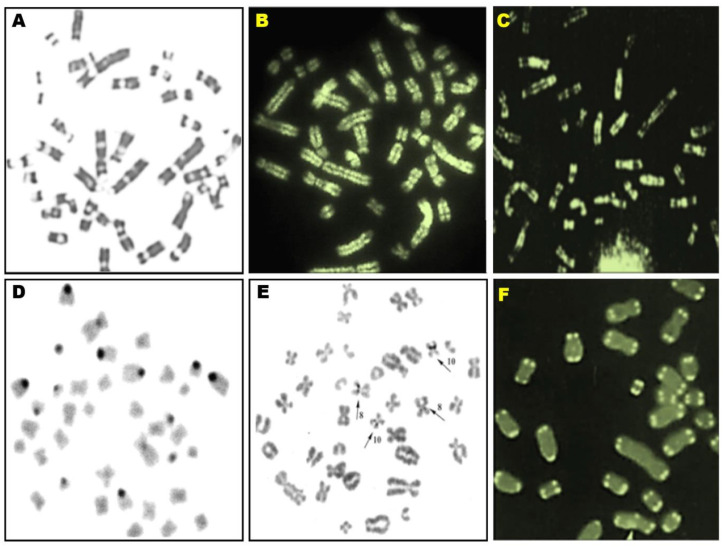
Pig metaphase chromosomes stained differentially using banding techniques G bands (**A**), Q bands (**B**), R bands (**C**), C bands (**D**), Ag-I bands (**E**), and T bands (**F**).

The next diagnostic stage is differential banding (resolution 5–10 million base pairs), which identifies 19 pairs of homologous chromosomes based on the similarity of size, morphological type, and Q-, G-, and R-banding patterns at 5–10 Mb resolution. The basic banding methods are complemented by techniques that stain selective chromosome regions: constitutive heterochromatin, nucleolar organizer regions, and telomere regions (C, Ag-I, and T banding) [2,7,13,59,60]. This diagnostic procedure determines the somatic cell karyotype of a specific individual by comparing individual banding patterns on chromosomes with the species standard of 300 Q, G, and R bands in the haploid chromosome set that has been developed as the standard karyotype of the domestic pig [61] (Figure 2).

Standard cytogenetic analysis is extended with high-resolution banding techniques (HRBTs) (resolution 2–5 Mb), which yield 600 G- or R-bands or sub-bands in the haploid set of elongated prometaphase chromosomes (obtained using replication and condensation blockers), corresponding to the standard karyotype for prometaphase chromosomes of the domestic pig [62,63].

In cases of suspected karyotype abnormalities (requiring a detailed assessment of biological consequences), classical cytogenetic diagnostics is complemented with additional laboratory procedures, which encompasses an analysis of the meiotic process in gonadal tissues (observation of conjugation processes in pachytene of prophase I and segregation in metaphases I and II under a light or electron microscope) [6,11,21,22,23,47,64,65].

In recent years, an indispensable diagnostic tool used for complex between-chromosome rearrangements and intrachromosomal micro-rearrangements (inversions, deletions, duplications) or marker chromosomes is fluorescence in situ hybridization (FISH; resolution 0.5–10 Mb) using molecular probes with different specificity levels (painting probes for whole chromosomes and their segments or probes specific for subtelomeric, telomeric and centromeric regions, and specific chromosome loci) [10,66].

The FISH technique enables the detection of DNA or RNA sequences in the analyzed material using a labeled complementary probe. This procedure consists of three basic steps: denaturation of cytogenetic preparations and molecular probes, probe hybridization, and probe detection [2,7,8,66]. This method also allows for interspecific in situ hybridizations (Zoo–FISH), where human probes are used to diagnose genetic defects or karyotype changes in different species of animals [67,68,69,70,71,72,73].

Studies of the domestic pig most often use painting probes as well as probes obtained by cloning genomic DNA inserts from genomic libraries (cosmid probes with DNA inserts < 20–40 kb; bacterial probes with DNA insert sizes of 120–150 kb) [1,2,8,60,74,75]. For pig genome analysis using the FISH technique, the most useful probes are chromosome-specific painting probes generated by flow sorting of chromosomes and PCR amplification of isolated DNA (DOP–PCR, PARM–PCR), as well as through needle or laser microdissection of chromosomes or their segments. [2,8,46,76,77,78,79,80,81,82].

The set of currently available painting probes for *Sus scrofa* includes flow-sorted probes for chromosomes 1, 3, 7, 13, 18, X, Y, and for the whole karyotype; probes obtained by microdissection for chromosomes 13, 15, 1p, chromosome-specific for bands 4p, 4q, and for all chromosomes; commercial probes X:Y (mix) and X and Y [8,68,77,78,79,80,81,83,84,85]. Additionally, oligonucleotide probes (primers) used in the PRINS technique (primed in situ labeling), specific for repeated DNA sequences, namely, telomeric, centromeric in acrocentric chromosomes and metacentric chromosomes (1, 2, 3, 4, 6, 7, 8, 9, 11, and X), as well as chromosomes (1, 9, 11, 14, Y), are available [8,86,87,88,89].

Recently, attempts have been made to detect unbalanced chromosomal aberrations using CGH (comparative genome hybridization) and array–CGH (microarray-based genome hybridization) methods, which identify sex chromosome aneuploidies and chromosome segments that have undergone amplification or deletion [50,90,91,92,93].

Cytogenetic monitoring based on the methods presented above is a significant element of the selection and prevention of chromosomal defects in pig breeding herds. The effectiveness of assessing pig karyotypes to find carriers of chromosomal mutations (mainly reciprocal translocations) is largely determined by the analytical screening procedures. The standard is banding methods, most often the GTG technique (G- banding method using trypsin for selective denaturation of protein structures and dye blocking) that yields chromosome-specific bands with at least 5 Mb diagnostic resolution [1,59,60,61]. A much higher experimental potential is offered by a multiprobe screening assay (developed in 2017) involving fluorescence in situ hybridization (FISH) of a panel of BAC (bacterial artificial chromosome) probes (specific for subtelomeric regions of the p and q arms), which enables precise and rapid identification of all chromosomes in one microscopic preparation [1,8,31,94]. Preliminary research has shown that this method is highly useful for diagnosing submicroscopic chromosomal rearrangements (impossible to detect with common cytogenetic tools), which may increase the detectability of reciprocal translocations, of which the frequency in the populations is probably much higher than previously estimated. For this reason, it seems especially justifiable to use this research tool prophylactically to assess the karyotypes of elite boars in AI centers (Table 1) [7,14,31,94].

## 4. Cytogenetic Screening of the Pig Population

Balanced chromosomal mutations, in particular reciprocal translocations associated with a drastic reduction of pig fertility, are seen as a major breeding and economic problem. Owing to this, many years ago, some countries introduced concrete selection guidelines to screen the karyotype of animals characterized by low breeding soundness or developmental abnormalities [4,12]. One example is the Scandinavian countries of Sweden and Finland, where numerous cases of reciprocal translocations were identified in the early 1990s as part of karyotype screening of animals selected based on breeding records that indicated a low number of piglets per litter (5 to 7 piglets) [64,95]. This allowed for the determination of the hypothetical frequency of the mutation carriers in the group of boars with reduced fertility (50%) and the estimation of the financial losses of USD6000 and USD25,000 incurred in one herd by sires carrying two different translocations [64]. Moreover, single cases of reciprocal translocations were identified during that period in pig populations raised in countries where no regular karyotype screening was conducted as part of national breeding evaluation programs, the result of which animals for cytogenetic tests were selected randomly or based on breeding recommendations associated with suspected developmental anomalies. One example is the reciprocal translocation that causes a slight decrease in fertility (5%), which was detected as part of pig herd monitoring in Germany (in the former GDR). In this case, the direct losses related to annual boar reproduction were estimated at around DDM28,000, and the global costs resulting from putting the offspring into breeding were much higher due to the accumulation of a small individual effect in a large population [39,95].

During the same period, at least several dozen new translocations were reported in France, where pig karyotype screening was included in the national system for the selection of breeds, lines, and individuals for high fertility [39,96]. As part of this program, in which the criterion was litter size, 800,000 litters sired by 20,000 boars were annually evaluated, and sires that produced fewer than 8 piglets in 6 successive litters were sent for cytogenetic analysis. In France, 42% of the boars with reduced fertility were then found to carry reciprocal translocations. In turn, assuming that the frequency of the boars with reduced fertility in the sire group was 0.15%, the frequency of the translocation carriers in the boar population was calculated to be 0.06% (one case per 1500 animals) [12,28,39]. Furthermore, in the 1990s, the PROSIM simulation model was used in France to analyze the economic consequences of reciprocal translocations using the example of a mutation that reduces fertility in the affected individuals by 45%. As part of this evaluation, financial outlays and revenue were compared in two equal herds of sows, one being mated to a translocation-carrying boar and the other to a boar with a normal karyotype. The direct farm losses from the failure to produce piglets (based on 65% mating success) were estimated at USD6000, and the losses resulting from the use of a translocation-carrying sire at the AI station was estimated to be USD105,000 (based on 650 semen doses per boar) [12,39,97]. In the United States, it was concluded based on estimates that the proportion of boars with low fertility was 3.7% (around 25 times that in France), which suggests the likelihood of a large number of translocation carriers. Therefore, in the 1990s, the US breeding program used the central boar selection system PIG CHAMP, based on similar assumptions as in France [39,95].

In other countries such as Hungary and The Netherlands, efforts were mainly concentrated on the cytogenetic screening of AI boars. As a result, several translocations were detected, and the frequency of chromosomal rearrangements among sires from Dutch AI centers was estimated to be 1.5%, which at that time was in excess of the expected value reported in the literature [4]. Additionally, in France, preliminary karyotype screening of 450 station boars showed the actual frequency of structural aberration carriers to be around 0.40%, much more than the hypothetical value reported earlier (0.06%), which was a strong argument for the intensification of screening tests when qualifying the boars for reproduction [4,29].

Additionally, in Poland, pig karyotype screening was not subject to any breeding rules, and such analyses (since the 1990s) could be performed only as part of boar reproductive performance tests. During that time, several hundred pigs that were randomly chosen from the population (using no fertility data, which are an indication for karyotype assessment) were subjected to screening, including only several dozen breeding and AI boars. This screening revealed the first reciprocal translocations that reduced fertility, the most fateful of which was translocation t(7;13)(q13;q46), which reduced the mean number of piglets per litter by 48% [12,39,67]. The simulated calculation of the economic consequences of carrying this defect showed that the financial losses caused by using one carrier boar in a herd are around USD8000 for natural mating, and USD162,000 for artificial insemination in the active population. These estimates are based on the loss of profit on a pig farm with an annual production of 3380 heads, or the loss of gross trade value from the sale of 1560 pigs, or the loss due to mortality of 11,267 day-old piglets [12,39].

The monitoring results from the 1990s encouraged other breeding centers to launch cytogenetic testing of boars from the reproductive sector. In Spain, based on the initial results of these analyses, the frequency of chromosomal abnormalities and structural anomalies in a commercial herd of more than 700 sires was estimated to be 3.8% and 3.3%, respectively [33,34,48,57,98,99]. In Canada, the frequency of karyotype defects among almost 900 boars qualified for reproduction in 2016 was estimated at 1.64% (with 1.36% animals carrying a translocation). Extrapolating this to a commercial scale, it was estimated that aberration-carrying piglets would occur in over 46,400 litters (out of around 2.9 million litters produced per year), which means an average loss of 4 piglets per litter. With the price of 25 CAD per piglet, this gives an annual loss of CAD4.6 million [16,35,41]. The results of this prediction analysis formed the basis for including almost 6000 boars (from Canadian breeding centers) in the program for cytogenetic screening and eradication of aberration carriers, which, in 2018, reduced the incidence of chromosomal mutations down to 0.91% [17,100].

The gradual expansion of artificial insemination in pig reproduction has increased the interest in cytogenetic monitoring of young boars qualified for reproduction in breeding centers. In some countries, considering the consequences of the high incidence of reciprocal translocations and the magnitude of financial losses, it was decided that systemic solutions for karyotype screening of all young boars before their use in AI stations would be adopted [4]. These activities are exemplified by the launching, more than 20 years ago in France, of a commercial cytogenetic platform at the l’Ecole Nationale Vétérinaire in Toulouse (ENVT–INRA), certified with the ISO 9001 standard since 2012, which allows large-scale monitoring of French pig populations [13,101,102]. The basic principle of the platform is to monitor the karyotype of young purebred boars and terminal crosses (aged 6 to 10 months) prior to use in AI stations at the request of breeders associations [4,13,15,27,28,29]. It should be noted that this laboratory concurrently performs cytogenetic screening of sires with reduced fertility parameters, and before that, it performed similar tests for the needs of breeding centers from other European countries (The Netherlands, Belgium, Germany, Spain) [13,27]. The effectiveness of the platform is evidenced by the data published in 2016–2018, according to which 39,000 boars were screened (up to 2000 per year) and over 180 cases of structural chromosomal abnormalities were identified [13,32,101]. Among the diagnosed aberrations, reciprocal translocations formed the overwhelming majority (87%), followed far behind by inversions (10%), Robertsonian translocations (2%), and other structural anomalies (1%). Apart from the structural rearrangements, chromosome aneuploidies, XX/XY cell chimerism, and sex-reversal cases were also diagnosed. For many years, the frequency of balanced chromosomal mutations has remained almost unchanged at around 0.5% (including reciprocal translocations 0.46%), and the studies did not reveal any breed trends in relation to the incidence of these changes, which confirmed their random nature [13,32,101]. As this type of rearrangement can generally lead to serious reproductive disorders (considerable reduction in litter size or infertility), it can be assumed that the ENVT–INRA program (operated in France since 1997) has saved pig producers from huge economic losses over the last 20 years. Additionally, in Poland, over the last 10 years, cytogenetic screening has included a group of several hundred young boars qualified for breeding, and the incidence of structural chromosomal rearrangements in this population, similar to France, was determined to range from 0.46% to 0.47% [12].

Recently, several European countries (The Netherlands, Spain, Sweden) that have intensified pig production through artificial insemination (using semen doses from a single sire) have emphasized the importance of boar karyotype screening as one of three components of an additional package of tests qualifying young boars for reproduction in AI centers. According to this concept, the estimated cost of implementing this package (in vitro fertilization test, assessment of nuclear chromatin, cytogenetic analysis) in an AI station with 100 boars would be around 100 euros per sire; this would allow an increase in mean litter size by 0.1 piglets and improve the economic effect by around 0.7 million euros [103].

It should be highlighted that the introduction of modern cytomolecular techniques into laboratory practice has considerably increased the diagnostic potential of the screening system, resulting in a marked increase in the number of karyotype abnormalities identified [92,103]. To date, cytogenetic screening of many pig populations around the world has resulted in the identification of over 220 structural karyotype defects, including almost 200 reciprocal translocations with a clear negative impact on fertility and economic efficiency of production [1,2,13,17,19,30,32,41,93,98,99,100,103,104,105,106,107]. It seems that the elaboration and implementation of the next screening strategies will significantly intensify the detectability of reciprocal translocations, which are likely much more frequent in breeding populations than previously estimated.

## 5. Conclusions

Cytogenetic diagnostics of pigs allow for effective selection that eliminates carriers of chromosomal aberrations from herds and thus reduces breeders’ financial losses, which are incomparably higher than the costs of routine karyotype testing.

The karyotype screening tests performed to date have confirmed the effectiveness of cytogenetic screening and the need for its continuation; they also fit well into the implementation of programs for sire assessment and selection, with special consideration of young boars qualified for insemination.

## Figures and Tables

**Table 1:** Cytomolecular diagnostics methods and the types of karyotype abnormalities.

Pig Karyotype Abnormalities	Effect on Carrier Fertility	Cytomolecular Diagnostics	Comments
Reciprocal translocations	Reduced fertility of sires and herds (5–100%)	Giemsa staining, differential banding techniques (GTG, RBA, QFQ), FISH	Techniques used for routine analysis: Giemsa staining, GTG technique, and FISH; in special cases, a multiprobe system for diagnostics of cryptic micro-rearrangement
Robertsonian translocations and tandem fusions	Reduced fertility (5–22%)	Giemsa staining, differential banding techniques (GTG, RBA, QFQ), FISH	Techniques used for routine analysis: Giemsa staining, GTG technique, and FISH in difficult cases
Peri- and paracentric inversions	Reduced fertility (less than 10%)	Giemsa staining, differential banding techniques (GTG, RBA, QFQ), FISH	Techniques used for routine analysis: Giemsa staining, GTG technique, and FISH in difficult cases
Sex chromosome aneuploidies and leukocyte chimerism	Reduced fertility or infertility	Giemsa staining, differential banding techniques (GTG, RBA, QFQ), FISH	Techniques used for routine analysis: Giemsa staining, GTG technique, and FISH with heterosome probes or the array–CGH method in difficult cases

GTG: G-banding method using trypsin and Giemsa; QFQ: Q-banding methods using quinacrine; FISH: fluorescence in situ hybridization; RBA: R-banding method using Giemsa and acridine orange.
